# Comparison of Laryngeal Mask Airway and Endotracheal Tube Placement in Neonates

**DOI:** 10.21203/rs.3.rs-3136331/v1

**Published:** 2023-07-13

**Authors:** Kari Roberts, Amanda Wanous, Roland Brown, Kyle Rudser

**Affiliations:** University of Minnesota Children's Hospital; University of Minnesota; University of Minnesota; University of Minnesota

## Abstract

**Objective:**

We hypothesize that the time, number of attempts and physiologic stability of placement of an LMA would be superior compared to ETT.

**Study Design:**

Videotape and physiologic parameters of LMA (n = 36) and ETT (n = 31) placement procedures for infants 28–36 weeks gestation were reviewed.

**Results:**

Duration of attempts (32 vs 66 sec, p < 0.001) and mean total procedure time (88 vs 153 sec, p = 0.06) was shorter for LMA compared to ETT. Mean number of attempts for successful placement was fewer for LMA (1.5 vs 1.9, p = 0.11). Physiologic parameters remained near baseline in both groups despite very different degrees of premedication.

**Conclusion:**

Placement of an LMA required less time and fewer number of attempts compared to ETT. Physiologic stability of an LMA was maintained without the use of an analgesic and muscle relaxant. Use of an LMA is a favorable alternative to ETT placement for surfactant delivery in neonates.

**Trial registration:**

NCT01116921

## INTRODUCTION

For decades, endotracheal intubation, a procedure associated with adverse physiologic effects such as bradycardia^1,2^, fluctuations in blood pressure^1-7^, hypoxia^1,2,6,8-10^ and increases in intracranial pressure ^2,3,5,7,11,12^, has been the gold standard for surfactant administration in neonates. However, with increased use of non-invasive ventilation, there has been an increased focus on less invasive methods of delivering surfactant to neonates with respiratory distress syndrome (RDS). This study is one component of a multicenter, randomized controlled trial investigating the use of a laryngeal mask airway (LMA) for surfactant administration in neonates^13^. This component investigates the physiologic stability during device placement and time and number of attempts required for successful placement of an LMA compared to placement of an endotracheal tube (ETT). We hypothesize that physiologic stability would be similar despite very different degrees of premedication and time and number of attempts would favor LMA.

## METHODS

Subjects were recruited at the University of Minnesota Masonic Children’s Hospital in Minneapolis, MN, St. Paul Children’s Hospital in St. Paul, MN, University of California San Diego Medical Center in San Diego, CA, Loma Linda University Medical Center in Loma Linda, CA, North Memorial Medical Center in Robbinsdale, MN, Maple Grove Hospital in Maple Grove, MN, and the University of Wisconsin- Madison Meriter Hospital in Madison, WI. Infants were 28 0/7–35 6/7 weeks gestation, ≥1250 grams and ≤36 hours old. Neonates with clinical or radiographic diagnosis of RDS requiring nasal continuous positive airway pressure (nCPAP) with supplemental oxygen (FiO2) of 0.30–0.40 were eligible for enrollment. Exclusion criteria included prior surfactant administration or mechanical ventilation, presence of congenital anomalies, or respiratory distress secondary to another condition (e.g., pneumothorax, pneumonia, meconium aspiration, etc.). Infants in the LMA Group had an LMA placed for surfactant administration. Infants in the Control Group did not receive surfactant. Infants in both groups who met treatment failure criteria were intubated. Clinical Trial Registration: clinicaltrials.gov ID NCT01116921

### Study Protocol

This study was performed in accordance with the Declaration of Helskinki, was approved by the Institutional Review Boards of all participating hospitals, and guardians had given written informed consent. Infants were randomized to LMA or Control groups stratified by center and gestational age (28–31 6/7 and 32–35 6/7 weeks). The LMA Group underwent LMA placement and surfactant administration with return to nCPAP following the procedure. The Control Group remained on nCPAP without surfactant administration. Treatment failure criteria was the same for both groups and required 2 of the following: (1) FiO2 > 0.40 for > 30 minutes (to maintain oxygen saturation (SpO2) 88%-92%), (2) pCO2 > 65 mmHg on arterial or capillary blood gas or > 70 mmHg on venous blood gas, or (3) pH < 7.22; or 1 of the following: (1) recurrent or severe apnea, (2) hemodynamic instability requiring pressors, (3) repeat surfactant dose, or (4) deemed necessary by medical provider. Infants who reached treatment failure criteria in either group in the first 7 days of life were intubated, treated with surfactant via the ETT and placed on mechanical ventilation.

Procedures (LMA placement for infants randomized to the LMA Group and ETT placement for infants in both groups who reached treatment failure criteria) were recorded using a custom designed data acquisition system. The system simultaneously recorded video information and analog physiologic data (heart rate (HR) and SpO2). Time-stamped digital video data was obtained with a video camera (Logitech Webcam C210) and analog signals from the oximeter (Radical, Masimo Corp, Irvine, CA) were processed through a MP150 data acquisition and AcqKnowledge software program (BioPAC Systems Inc, Goleta, CA). Neonatal clinicians, which included neonatologists, neonatology fellows and neonatal nurse practitioners, had little or no prior experience with LMA placement. Training occurred through demonstration and practice on a manikin. The same group of clinicians had experience in intubation and did not receive additional training for ETT placement.

For LMA placement, neonates were premedicated with 24% oral sucrose solution (Sweet-Ease^®^, Philips, Atlanta, Georgia) (1 mL to the tip of the tongue) and atropine (0.02 mg/kg IV over 1 minute). An LMA (LMA Unique, Size 1, LMA North America, Inc, San Diego, CA) was inserted until the provider was unable to advance further and the cuff was inflated with 3 cc of air.

For ETT placement, neonates were premedicated with atropine (0.02 mg/kg IV), fentanyl (2 mcg/kg IV), and rocuronium (0.6 mg/kg IV). An ETT was placed using the appropriate size laryngoscope and ETT once muscle relaxation occurred.

For both LMA and ETT placement procedures, successful placement was confirmed with CO_2_ colorimeter (PediCap, Nellcor Puritan Bennett, Pleasanton, CA). If yellow color change was not observed, the device was repositioned or removed. Placement attempts were discontinued if SpO2 < 75%, HR < 100 beats per minute (bpm), or if the duration of the attempt exceeded 30 seconds, even if the infant remained stable. If more than one attempt was required, bag mask ventilation was administered and a repeat attempt was initiated once SpO_2_ ≥ 95% and HR > 100 bpm.

HR and SpO_2_ change from baseline were analyzed as measures of physiologic stability during placement. Baseline values were obtained for thirty seconds prior to initiation of the first attempt.

Videotape of LMA and ETT placement were reviewed to determine the time and number of attempts required for successful placement. An individual attempt was defined as insertion of the LMA (LMA Group) or laryngoscope (ETT Group) into the mouth. Duration of an attempt was defined as the time from insertion of the LMA into the mouth through inflation of the cuff (LMA Group) or insertion of the laryngoscope through removal of the laryngoscope (ETT Group). Total procedure time was defined as the duration from first insertion of the LMA (LMA Group) or first insertion of the laryngoscope (ETT Group) until proper placement was confirmed (includes all attempts and bag-mask ventilation between attempts).

Study data were maintained using REDCap electronic database hosted at the University of Minnesota^14^. All statistical analyses were conducted using R v3.6.1^15^. Continuous variables were summarized with averages, standard deviations, medians and range, including change from baseline values for physiologic outcomes. Differences in mean outcomes between ETT and LMA groups were estimated using linear regression. Relative risks for binary outcomes were estimated using log-binomial regression. Robust variance estimation was used when calculating confidence intervals and p-values. HR and SpO2 data were analyzed as change from average baseline values to average values during procedural time.

## RESULTS

**Figure 1:**
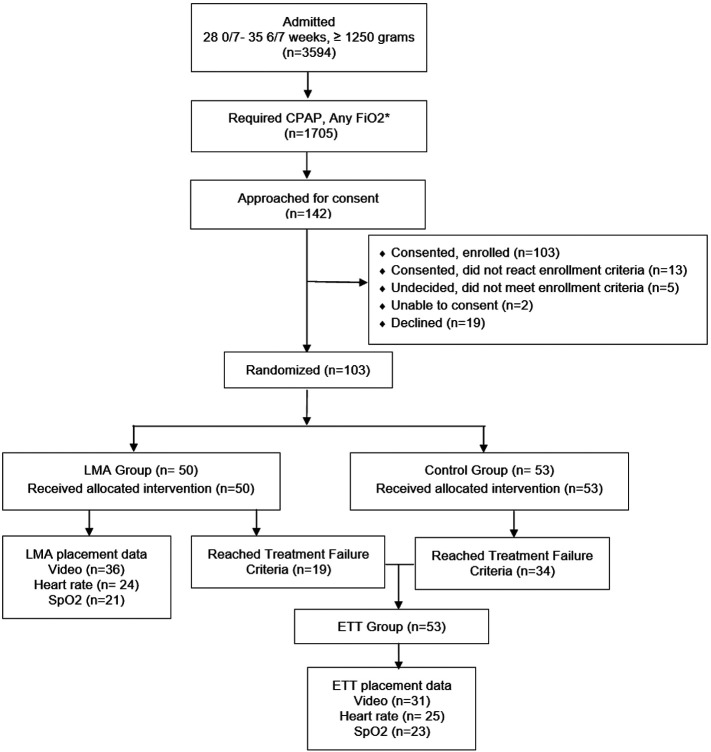
CONSORT diagram *Data unavailable for FiO2 0.30-0.40 for > 30 minutes

During the study period from February 2011- April 2015, 103 neonates were enrolled (LMA Group n=50, Control Group n=53). A total of 53 infants (LMA Group n=19, Control Group n=34) reached treatment failure criteria and were intubated. Due to technical difficulties, data were not captured on all enrolled infants. In the LMA and ETT groups respectively, videotape of the complete placement procedure were available for 36 and 31 neonates, HR data were available for 24 and 25 neonates, and SpO_2_ data were available for 21 and 23 neonates (Figure 1). Baseline characteristics of participants are shown in Table 1.

Physiologic parameters remained near baseline in both LMA and ETT groups with HR change +1 bpm and −1 bpm (p=0.33) and SpO_2_ change −7% and −4% (p=0.36), respectively.

**Figure 2:**
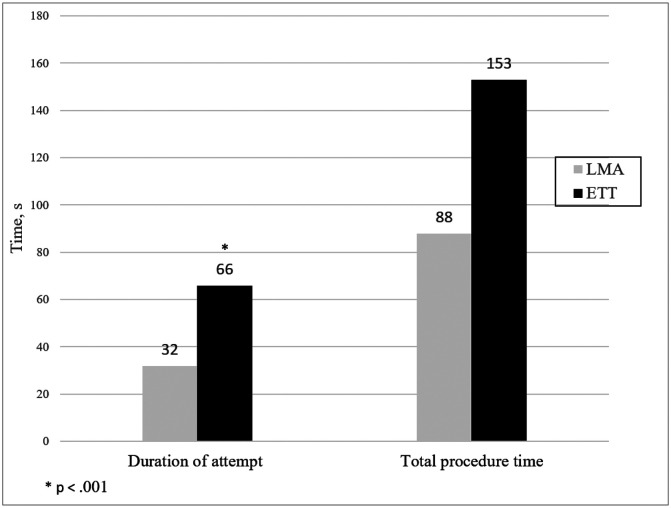
Time Results

Average duration of attempts and mean total procedure time are shown in Figure 2. Number of attempts required for successful placement of the device are shown in Table 2.

## DISCUSSION

Our study showed that physiologic stability during placement of an LMA was maintained close to baseline without use of an analgesic and muscle relaxant. In addition, duration of attempts was shorter for LMA compared to ETT placement. The mean total procedure time and mean number of attempts for successful placement favored LMA placement although were not statistically significant.

Intubation is a skill that is difficult to learn, has a low first attempt success rate, and is associated with adverse events. In a review of 701 intubations in a single- center, Level 4 NICU, Foglia et al^16^ found an adverse event rate of 22%, with the most common events being esophageal intubation (16%), mainstem intubation (2%), oral/airway trauma (3%), vomiting (1%) and cardiac arrest (1%). In a review of 273 intubation attempts in 162 infants in a single-center, Level 4 NICU, Hatch et al^17^ found an adverse event rate of 39%. Similar to the Foglia data, the most common adverse events were esophageal intubation (21%), oral/airway bleeding (9%), mainstem intubation (7%) and hypotension (4%). In 2019, the National Emergency Airway Registry for Neonates (NEAR4NEOS), a multicenter registry of neonatal intubation, published data on 2009 NICU intubations. This study reported that 18% involved at least 1 adverse event^18^ and a severe adverse event (defined as: cardiac arrest, esophageal intubation with delayed recognition, hypotension requiring therapy, cardiac compressions lasting < 1 minute, laryngospasm, pneumothorax, pneumomediastinum, and/or requiring transition from non-emergent to emergent intubation) occurred in 4–9% of intubations^17,18^.

Intubation is also associated with adverse physiologic effects, including bradycardia^1,2^, fluctuations in blood pressure^1-7^, hypoxia^1,2,6,8-10^, and increases in intracranial pressure ^2,3,5,7,11,12^. In recent clinical studies, severe desaturation, defined as SpO2 < 60% or a decrease in SpO2 ≥ 20% from baseline, occurred in approximately 45–65% of intubation attempts^16-20^.

In a recent editorial discussing the hazards of intubation, the author states: “These factors conspire to make intubation one of the most dangerous procedures in neonatal medicine^21^.”

In our study, infants in the LMA group received atropine and 24% sucrose solution while the ETT group were premedicated with atropine, fentanyl and rocuroium. Physiologic parameters were maintained close to baseline during LMA and ETT placement. Atropine was used as premedication in both groups, therefore minimal change in HR was not unexpected. What is notable, however, is change in SpO2 was similar between groups, given the dramatic difference in degree of premedication.

Minimal change in baseline SpO2 without use of an analgesic and muscle relaxant in the LMA Group is an important finding. Previous studies have shown that premedication prior to intubation with a muscle relaxant decreases the incidence of severe desaturation. A study investigating the use of premedication with and without a muscle relaxant (atropine, fentanyl compared to atropine, fentanyl and mivacurium) in non-emergent intubations in the NICU found that use of a muscle relaxant significantly decreased the incidence of severe desaturation (SpO2 ≤ 60%) (55% vs 24%, p = 0.041)^20^. Similarly, in an intervention study with the aim of improving patient safety during intubations, implementation of premedication resulted in a significant reduction of intubation associated adverse events (46% vs 36%, p = 0.02) and incidence of severe hypoxemia (SpO2 < 60%) (44% vs 33%, p = 0.006)^19^.

The difference in degree of premedication between the two groups is an important distinction, especially for centers that do not routinely use analgesics and/or muscle relaxants prior to intubation, as using an LMA may provide *greater* physiologic stability during the procedure compared to intubation without premedication. The LMA may also provide greater physiologic stability compared to the INSURE technique (Intubation, Surfactant administration and Rapid Extubation) for surfactant administration. With the INSURE technique, use of an analgesic has been shown to result in increased extubation failure^22^ and a paralytic is not appropriate given the need to remain spontaneously breathing.

Our study also showed that successful placement of the device favored the LMA, as LMA placement requiring less time and fewer number of attempts compared to placement of an ETT. Again, the difference in degree of premedication is notable. A study investigating the use of premedication with and without a muscle relaxant (atropine, fentanyl compared to atropine, fentanyl and mivacurium) found that use of a muscle relaxant decreased the time (472 vs 144 seconds; p = .003) and number of attempts needed to successfully intubate (successful in ≤ 2 attempts, 35% vs 71%, p = 0.019)^20^. Similarly, in the NEAR4NEOS registry, use of a muscle relaxant was associated with a significant decrease in difficult intubations (50% vs 23%, p < 0.001)^23^. In our study, had intubation occurred without the use of a muscle relaxant, time, number of attempts and incidence of severe oxygen desaturation in the ETT group may have been greater than what was seen.

A strength of this study is the generalizability of the findings, as the study was conducted at seven academic teaching hospitals. Providers were neonatologists, neonatal-perinatal fellows and neonatal nurse practitioners skilled in intubation, and had little experience in placing an LMA. Our study showed that even with little or no previous experience, placing an LMA was faster and easier than placing an ETT.

A limitation of the study is the technical issues with the recording equipment which precluded complete video and physiologic information for all neonates enrolled in the study.

Due to the ease of use, ability to place with minimal equipment, and no need for a mechanical ventilator, use of an LMA has application not only in high-resource settings, such as Neonatal Intensive Care Units, but also may have significant benefit for infants in community hospitals in high-income countries as well as infants born in low- and middle-income countries.

## CONCLUSION

Physiologic stability during device placement was similar between LMA and ETT groups despite very different degrees of premedication, with LMA placement receiving atropine and 24% sucrose solution while the ETT group received atropine, an analgesic and muscle relaxant.

Duration of placement attempts was 50% shorter for LMA placement and mean total procedure time and mean number of attempts for successful placement, while not statistically significant, favored LMA placement. These data suggest considering LMA use over ETT based on device placement characteristics.

## Figures and Tables

**Table 1.** Baseline characteristics

Variable	LMA Placement (n=36)	ETT Placement (n=31)
Gestational Age, mean (SD) (range), weeks	33° (2)	33° (2)
	(29^3/7^-35^4/7^)	(28^5/7^-35^6/7^)
Birth weight, mean (SD) (range), g	2006 (483)	2059 (531)
	(1290-3180)	(1254-3305)
Female, No. (%)	14 (39)	10 (32)
Baseline HR, mean (SD) (range), bpm	166 (15)	169 (21)
	(137-191)	(121-217)
Baseline SpO_2_, mean (SD) (range), %	91 (8)	89 (12)
	(74-99)	(50-100)

**Table 2.** Number of attempts required for successful placement

Number of Attempts			
Variable	LMA	ETT	p
No. of attempts, mean (range)	1.5 (1- 4)	1.9 (1-6)	.066
Attempts = 1, No. (%)	25 (69)	18 (58)	.292
Attempts ≤ 2, No. (%)	30 (83)	21 (68)	.127

## Data Availability

Descriptive analysis of placement of the LMA has been published in *Neonatology* 2016; 111(3): 222-227. DOI 10.1159/00045069. *Deidentified individual participant data (including data dictionaries) for LMA and ETT data will be made available, in addition to study protocol, the statistical analysis plan, and the informed consent form. The data will be* made available upon publication to researchers who provide a methodologically sound proposal for use in achieving the goals of the approved proposal. Proposals should be submitted to rober694@umn.edu.
